# Determining nitrogen isotopes discrimination under drought stress on enzymatic activities, nitrogen isotope abundance and water contents of Kentucky bluegrass

**DOI:** 10.1038/s41598-020-63548-w

**Published:** 2020-04-14

**Authors:** Shah Saud, Shah Fahad, Guowen Cui, Chen Yajun, Sumera Anwar

**Affiliations:** 1 College of Horticulture, Northeast Agricultural University Harbin, 150030 Heilongjiang, China; 2 Department of Agriculture, University of Swabi, Khyber Pakhtunkhwa, Pakistan; 3 College of Plant Science and Technology, Huazhong Agricultural University, Wuhan, Hubei 430070 China; 4 College of Animal Sciences and Technology, Northeast Agricultural University Harbin, 150030 Heilongjiang, China; 5 Institute of Molecular Biology and Biotechnology. The University of Lahore, Lahore, Pakistan

**Keywords:** Plant sciences, Environmental sciences

## Abstract

Drought stress is the most pervasive threat to plant growth, which predominantly encumbers turf grass growth by causing alterations in plant functions. This study appraised the role of nitrogen isotopes in providing a theoretical basis for developing and improving Kentucky bluegrass cultivar performance under drought stress. Nitrogen isotopes labelled ^15^NH_4_Cl and K^15^NO_3_ were prepared to replace KNO_3_ in Hoagland’s solution at concentrations of ^15^NH_4_^+^ and ^15^NO_3_ at 1.5, 15, and 30 mM; the solutions were imposed on stressed plants under glasshouse conditions. Nitrogenous nutrition reduced oxidative stress by elevating the enzymatic activities and proline contents of all three clonal ramet leaves, particularly under stress conditions. Apart from nitrogen content, nitrogen isotope abundance, relative water content and water potential within controls were enhanced in treated with ^15^NH_4_^+^ than in with ^15^NO_3_ in both the roots and leaves of Kentucky bluegrass. Nevertheless, an application of ^15^NH_4_Cl and K^15^NO_3_ at 30 mM had a positive influence to some extent on these attributes under drought stress. Overall, our results suggested that nitrogen isotopes contributed to drought tolerance in all three clonal ramets of Kentucky bluegrass by maintaining a better osmoprotectant and antioxidant defence system, which helped the plants eliminate reactive oxygen species.

## Introduction

Climate change models predict that rising atmospheric CO_2_ partial pressure during the next 100 years will cause average surface temperatures to rise by 1–3.5 °C in mid-latitude regions^1,2^. As a result, changes will occur in precipitation and evaporation patterns, and ecosystems will be exposed to more frequent drought events^2,3^. Furthermore, a persistent aridity enlargement has been recorded since the middle of the 20th century, and according to present projection models, this process will continue^4^. In some regions where a declination is predicted in crop performance, many advances have to be developed in plant breeding programmes and agricultural technology^5^. Understanding the adaption and response of drought and their mechanisms is essential for the achievement of those aims.

In water scarce areas, drought stress is considered one of the most devastating abiotic stresses for turf grass performance. Water stress happens in the crop either when the supply of water to the roots is intermittent or when the rate of transpiration goes beyond their optimum range, and under arid and semi-arid climate regions, these two conditions frequently coincide. The circumstances become further intensified due to the receding water table and mounting global warming. Plant adaptive strategies to drought stress are very multifaceted but not mutually exclusive, in practice, plants may combine a range of response types^6^. Literatures have addressed many important physiological and biochemical mechanisms of plants resistance to drought, such as the role of antioxidant enzymes, osmotic adjustment, membrane proteins mediating ions transports^7–12^. Often in plants, these detoxification (SOD, POD, CAT), osmoprotection (proline, sugar), ions movement and genes regulation are synergistic resistance to drought resistance^13^. In fact, plants suffered water deficit induce a decrease in nutrients assimilation, in particular nitrogen, with strong interactive effects on plant growth and performance^14^.

Some clonal plants^15^ with unique ramets system by interval connected to each other adapt to severe environment through phenotypic plasticity and physiological integration allowing water and nutrients transfer and redistribution between the clonal ramets. During nutrient transport process, water is the main carrier. However, the directions and the extent of the integration of water and nutrients physiology of clonal plants are different^16^. Kentucky bluegrass (*Poa pratensis* L.) is a typical clonal plant which spreads by rhizomes and tillers and produces many ramets in its clonal system^17^. It is also an excellent perennial cold season turfgrass. When temperatures are optimal (18 to 24 °C), along with an adequate amount of water and proper nutrient management, it forms an attractive turf; nevertheless, rapid colour loss, thinning, and dormancy are observed during periods of prolonged heat and water stress situations^18^. Water scarcity, along with low nitrogen (N), is an important constraint to Kentucky bluegrass performance, and has broadly been documented in influencing leaf water relations and photosynthetic attributes that result in decreased plant growth rate, early senescence, and poor plant efficiency^10,19–21^. An appropriate amount of nitrogen can have a significant effect on controlling turf grass density and weeds, and it is good to extend the turf grass green period and advance the re-greening period^22^. Leaf width and length are determined by genetic genes, but the appearance quality of turf grass is correlated with the amount of nitrogen fertilizer^23^ In general, former studies focus on individual plant, information on nutrients translocation and physiological responses to drought stress between ramets system of Kentucky bluegrass is lacking. Understanding the physiological adaptation of clonal ramets system of Kentucky bluegrass to drought stress may provide improved selection traits for breeding programs, leading to cultivars with good clonal attributes based drought resistance.

An adequate evaluation of drought stress influence under different N isotopic levels on physiological attributes can provide a better understanding of Kentucky bluegrass under drought stress^24^ Stable isotope methods have been utilized as tools that deliver valuable information on factors affecting plant growth, for instasnce, transpiration efficacy and the ratio of net photosynthesis to transpired water, among others, and those that integrate the duration through which CO_2_ is assimilated^25–27^. Variations in ^15^N isotopic composition (δ^15^N) have also been suggested as a beneficial feature for crop screening^28,29^. Robinson *et al*.^30^ documented that the natural profusion of ^15^N might play a vital role in the responses to numerous abiotic stresses such as drought and nitrogen starvation. Furthermore, δ^13^C and δ^15^N have been utilized to illustrate plant responses to salinity stress^31^ and are broadly used in plant ecophysiology to evaluate the consequences of varying climatic due to their sensitivity sensitive to environmental constraints^32^ In addition, variations in N source and the distribution of N assimilation between the roots and shoots are known to affect intra-plant changes in δ^15^N^33^. Therefore, assessing N isotopes in several plant parts can provide new insights into drought influences on nitrogen metabolism inside Kentucky bluegrass plants.

Currently, most studies related to Kentucky bluegrasses focus on nitrogen uptake, metabolism and utilization efficiency under conditions of no stress, and despite recent advances in the understanding of plant δ^15^N, there are no reports about the physiological integration of isotopic nitrogen in clonal populations of Kentucky bluegrass under heterogeneous ecological conditions. Therefore, the objectives of this study were (1) to determine the role of nitrogen tracers as an indicator of stress for Kentucky bluegrass, (2) to explore nitrogen isotopes influence on the physiological traits of ramets, and (3) to investigate the potential interference of nitrogen tracers on nitrogen isotope abundance and nitrogen content in both the roots and leaves of Kentucky bluegrasses, all of which can provide valuable information on the transport and metabolism of nitrogen under drought conditions.

## Materials and Methods

### Plant Material and growth conditions

Sod (10-cm diameter) of Kentucky bluegrass cv. “Arcadia” was collected from a turf field with a metal hollow drill on the sixth of June, 2014. Arcadia is a renowned, low water and nitrogen input cultivar and can acclimatize to the climatic and environment conditions in North China. Grasses were first grown in seedling trays (70 cm × 30 cm × 10 cm), and after 5 days of germination and with the appearance of first two leaves, seedlings were transplanted to pots. During this period, the average daily day and night temperatures were 25 ± 2 and 15 ± 2 °C, respectively. Relative humidity was 60 ± 5%, and natural sunlight at 700 ± 10 μmol m^−2^s^−1^ was maintained.

Three identical growing ramets were selected as the research materials from a genetically identical clone population from the early seedlings^34^. Fresh mass and morphological properties of the ramets (rhizome length and plant height) were determined before treatment. The ramets were classified as either a proximal ramet (PR) for the close distance between the ramet and the donor plant, as a distal ramet (DR) for having rhizomes, or as a middle ramet (MR), which is in the middle of the three ramets. Three plastic pots were prepared (length × width × height = 10 cm × 10 cm × 20 cm) and filled with fine sand to a height of 18 cm (particle size was between 0.2 and 1.0 mm). Subsequently, a slit was cut down from the edge of the pot and a hole was drilled at the very end of each slit. Three sub strains of plant material were planted into pots separately, which were connected through the holes via connected rhizomes and were sealed from neighbouring pots by glue to avoid water, sand and nutrient loss. Rhizomes were covered by 5 cm sand and surrounded by dark PE film to avoid sun light. Plants were irrigated by Hoagland’s solution every two days and then irrigated by deionized water periodically for the rest of the days.

### Treatments

**Figure 1:**
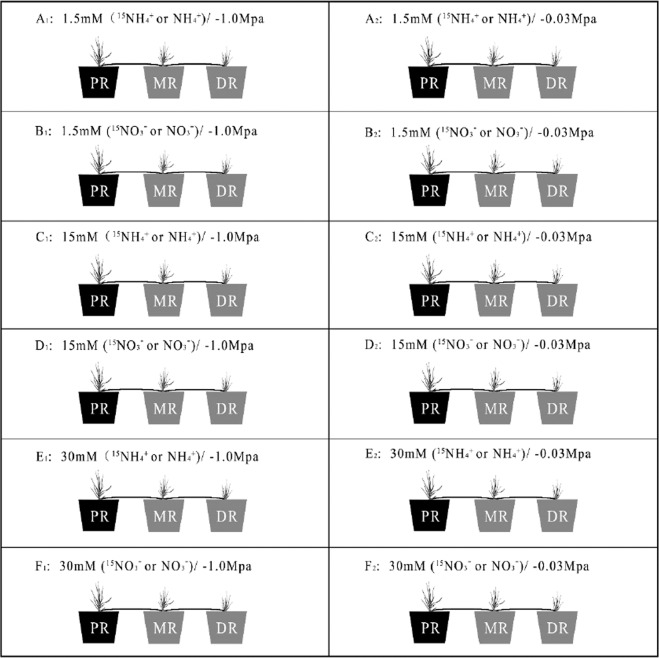
Schematic representation of the experimental treatments with three concentrations (1.5 mM, 15 mM and 30 mM) of nitrogen (^15^NH_4_^+^, NH_4_^+^, ^15^NO_3_^−^ and NO_3_^−^) under normal water (Osmotic potential of the solutions of A_1_, B_1_, C_1_, D_1_, E_1_ and F_1_ was −0.03 Mpa) and drought (Osmotic potential of the solutions of A_2_, B_2_, C_2_, D_2_, E_2_ and F_2_ was −0.1Mpa) conditions were imposed on Kentucky bluegrass ramets (PR, MR and DR). Solutions with ^15^NH_4_^+^or ^15^NO_3_^-^ were added into the black beakers (PR) in each group. Grey beakers holding with MR and DR in the same group were added with either NH_4_^+^or NO_3_^−^ in the same concentration. PR, MR and DR were connected by rhizome.

Nitrogen sources including NH_4_Cl, KNO_3_, isotopes (δ^15^N) labelled ^15^NH_4_Cl and K^15^NO_3_ were prepared using Hoagland’s solution at the concentrations of 1.5, 15, and 30 mM NH_4_^+^, NO_3_^−^, ^15^NH_4_^+^ and ^15^NO_3_^−^. For drought stress conditions, PEG6000 was added to the solutions and was adjusted to an osmotic potential of −1.0 Mpa, while the solutions of the controls without PEG6000 had an osmotic potential of −0.03 Mpa. The Root-DS and Leaf-DS represented the parameters of the roots and leaves under drought stress, and Root-CK and Leaf-CK as control groups represented the parameters under no drought conditions. Each treatment was taken in four replicates during whole experiment. After 2 weeks of cultivation, the three plants connected by rhizome were obtained by removing the sand and cutting the root bottoms without destroying the rhizome. The roots of three ramets were washed with distilled water, dried by filter paper, and cultured in three beakers (50 ml) separately as shown in Fig. 1.

The designated solution with δ^15^N at specific concentration was added into the beaker with PR, while the other two beakers holding with MR and DR in the same group were added with either NH_4_^+^or NO_3_^−^ in the same concentration. After 48 h treated rhizome connected with ramets system of PR, MR and DR, leaves and roots of these ramets were taken for parameters measurement.

### Data collection

#### N isotope and N contents analysis

The abundance of δ^15^N isotopes from the roots and leaf of different ramets were determined by EA-IRMS (EA: vario MICRO cube, Elemental, Germany; IRMS: IsoPrime100, Iso Prime, England). The roots and leaf materials were oven-dried and ground separately and were transferred into small tin capsules in an element analyser tray. They were oxidized into nitrogen oxide and CO_2_ at 950 °C, and afterwards, NO_X_ was reduced to N_2_ at 550 °C with CO_2_ absorbed on the desorption column, and N_2_ entered into the analyser system for analysis. Both the amount of N (expressed by mass percentage) and the abundance of isotopes were obtained. N content was also determined by the standard macro-Kjeldahl procedure using a Kjeltec 2300 analyser unit (Foss Tecator AB, Hoganas, Sweden). Both roots and leaf samples were ground to powder, weighed (∼0.1 g each) and then digested in H_2_SO_4_-H_2_O_2_.

#### Antioxidants

The activity of superoxide dismutase (SOD) was assayed following the method described by^35^ Peroxidase (POD) and Catalase (CAT) activities were also assayed following the method described by Bai *et al*.^35^. The 3 ml reaction solution for POD contained 50 mM phosphate buffer (pH 7.8), 25 mM guaiacol, 200 mM H_2_O_2_, and 0.5 ml enzyme extract. One unit of POD activity was determined as 0.01 units/min change in absorbance.

#### Relative water content (RWC), water potential (Ψw), and Proline

Relative water content (RWC) of both leaves and roots were determined based on fresh (FW), turgid (TW), and dry weights (DW) using the following formula: RWC (%) = [(FW − DW)/(TW − DW)] × 100. The leaf and root water potential (Ψw) were determined following the method of Bruggink and Huang^36^ by using a pressure chamber (PMS Instrument Co., Corvallis, OR, USA). While proline content was determined according to Gilmour *et al*.^37^.

#### Experimental design and statistical analyses

The experiment was carried out using a complete randomized design (CRD) with four replicates. Data were analysed by analysis of variance using SPSS v 9.0 software (SPSS, Inc., Chicago IL). The mean values were compared with a least significance difference test at a 0.05 probability level. Mean graphs were made using Sigma plot v.10 (Systat Software, San Jose, CA)^38^.

## Results

### Changes in catalase activity in the clonal ramets of Kentucky bluegrass under different nitrogen tracers and water supplies

**Figure 2:**
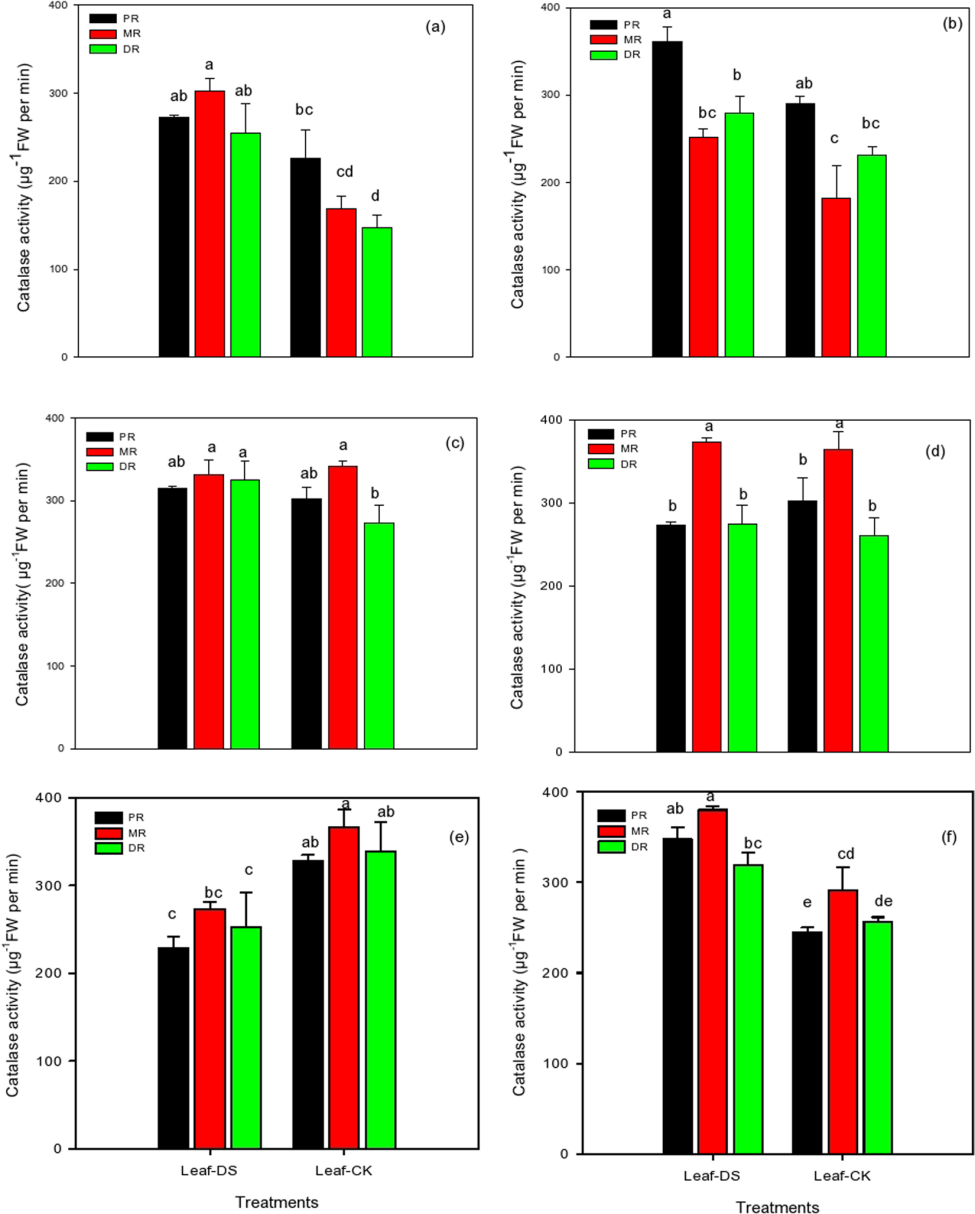
Catalase activity in the clonal ramets of Kentucky bluegrass as affected by drought stress under three nitrogen isotopes at low, moderate and high rates. (**a**) ^15^NH_4_Cl at 1.5 mM, (**b**) K^15^NO_3_ at 1.5 mM, (**c**) ^15^NH_4_Cl at 15 mM, (**d**) K^15^NO_3_ at 15 mM, (**e**) ^15^NH_4_Cl at 30 mM, and (**f**) K^15^NO_3_ at 30 Mm.

Drought stress under various levels of fertilization significantly affected the catalase activities of the proximal ramets (PR) and the distal ramets (DR) compared to the middle ramets (MR) in all treatments (Fig. 2). An application of NH_4_Cl at 1.5 mM showed an increase in PR, MR, and DR of 17%, 44%, and 42%, respectively; however, with KNO_3_, an increase of 20%, 28% and 17% was observed, respectively, compared to well-watered conditions (Fig. 2a,b). The fertilization of NH_4_Cl and KNO_3_ was beneficial under drought stress, and both fertilizers applied at 15 mM recorded an increase of 16% and 5% in DR, respectively, while in MR and PR, there was a 2% and 10% reduction in catalase activity with the KNO_3_ treatment compared to the control, respectively (Fig. 2c,d). Furthermore, it was noted that the catalase activity in all treatments of PR, DR, and MR under the high level of 30 Mm NH_4_Cl applications declined abruptly when Kentucky bluegrass was exposed to drought stress (Fig. 2e). The data pertaining to PR, MR, and DR indicated a reduction of 38%, 26% and 26%, respectively, under drought conditions. Among all three attributes, MR was the least influenced, followed by DR and PR. An increase in the catalase activity of Kentucky bluegrass was proportional to the rate of KNO_3_, and KNO_3_ at 30 mM recorded the highest activity (Fig. 2f). Drought stress enhanced the activity of all three attributes, as this treatment increased the activity of MR (35%), followed by PR (29%) and DR (20%). The overall fertilization of NH_4_Cl and KNO_3_ at 30 mM increased the catalase activity of all three attributes, as shown in Fig. 2.

### Changes in SOD in the clonal ramets of Kentucky bluegrass under different nitrogen tracers and water supplies

**Figure 3:**
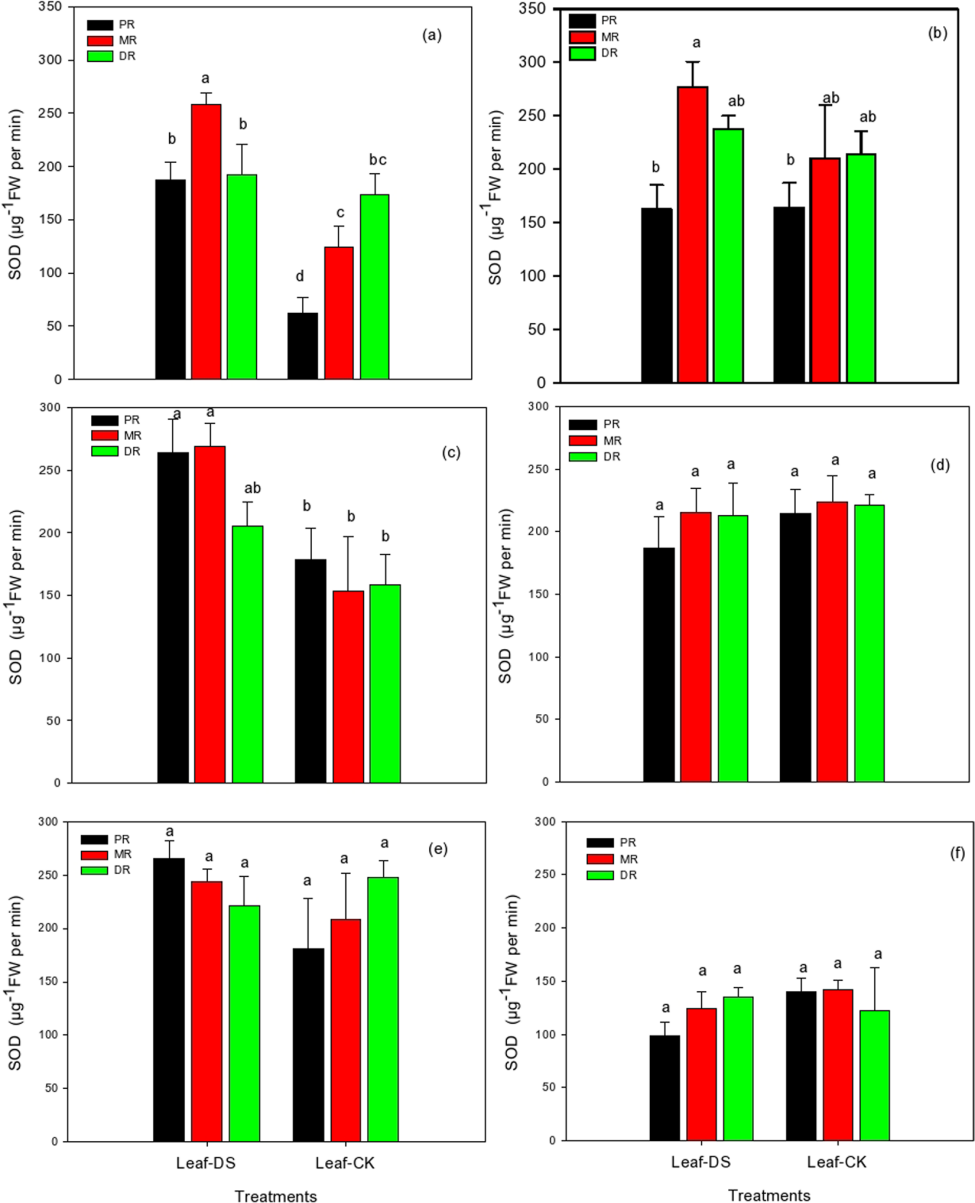
SOD activity in the clonal ramets of Kentucky bluegrass as affected by drought stress under three nitrogen isotopes at low, moderate and high rates. (**a**) ^15^NH_4_Cl at 1.5 mM, (**b**) K^15^NO_3_ at 1.5 mM, (**c**) ^15^NH_4_Cl at 15 mM, **(d**) K^15^NO_3_ at 15 mM, (**e**) ^15^NH_4_Cl at 30 mM, and (**f**) K^15^NO_3_ at 30 mM.

Superoxide dismutase (SOD) activity in Kentucky bluegrass was severely influenced by the fertilization of NH_4_Cl and KNO_3_ under drought stress conditions (Fig. 3). An application of NH_4_Cl and KNO_3_ at 1.5 mM significantly and linearly increased the activity of PR, MR, and DR; nevertheless, among the traits we examined, PR and MR in NH_4_Cl was increased 66% and 52%, respectively, while MR and DR in KNO_3_ had the highest SOD activity (Fig. 3a,b). Among the control, DR followed by MR had the highest activity. We addressed the impact of moderate levels of nitrogenous fertilizer on SOD activity in the three traits of Kentucky bluegrass. All PR, MR, and DR showed increased activity under drought stress at 15 mM of NH_4_Cl. MR followed by PR and then DR were the best, giving a maximum enhancement of 43%, 32% and 23%, respectively (Fig. 3c). An application of 15 mM of KNO_3_ remained effective in alleviating the adverse influence of drought stress but was not as effective as NH_4_Cl. Compared to the control, PR, MR, and DR recorded 13%, 4% and 3% less activity in Kentucky bluegrass, respectively (Fig. 3d). A non-significant effect was recorded with the high level of NH_4_Cl at 30 mM in all the three grass characters when the plants were subjected to water stress conditions. Among the three attributes, PR demonstrated an increase of 32%, followed by MR (15%), while on the other side, DR decreased by 27% (Fig. 3e). An application of KNO_3_ at 30 mM had a less pronounced effect on the activity of all three traits. The KNO_3_ treatment exhibited a 29%, 13% and 10% decline in the SOD activity of PR, MR, and DR, respectively, compared to the control treatment without a high dose of fertilizer (Fig. 3f).

### Changes in POD in the clonal ramets of Kentucky bluegrass under different nitrogen tracers and water supplies

**Figure 4:**
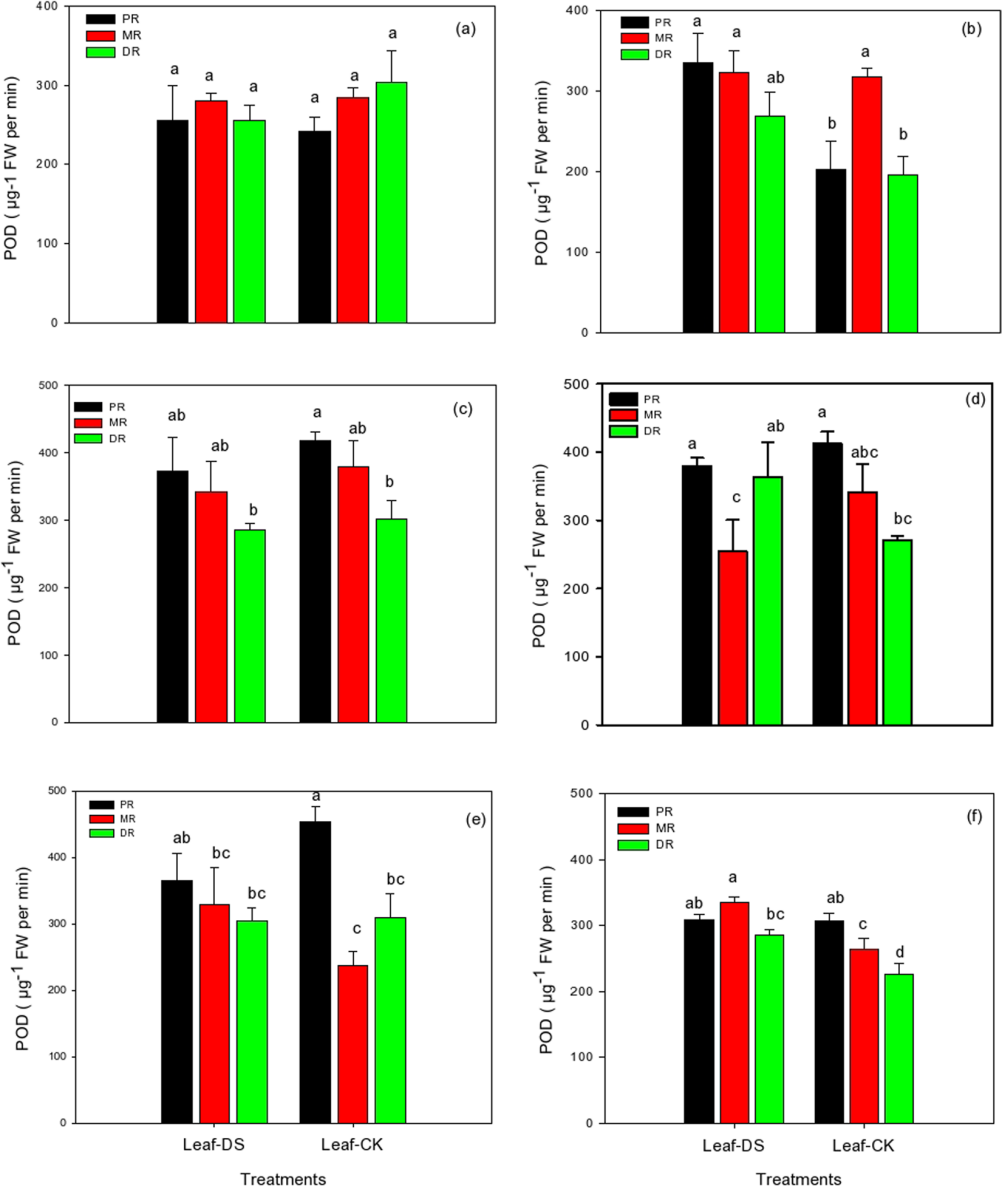
POD activity in the clonal ramets of Kentucky bluegrass as affected by drought stress under three nitrogen isotopes at low, moderate and high rates. (**a**) ^15^NH_4_Cl at 1.5 mM, (**b**) K^15^NO_3_ at 1.5 mM, **(c**) ^15^NH_4_Cl at 15 mM, (**d**) K^15^NO_3_ at 15 mM, (**e**) ^15^NH_4_Cl at 30 mM, and (**f**) K^15^NO_3_ at 30 mM.

The data regarding peroxidase (POD) activity of Kentucky bluegrass revealed that the activity of all three traits was influenced by the different levels of nitrogenous fertilizer during progressive drought stress (Fig. 4). A non-significant decrease was observed with the application of NH_4_Cl at 1.5 mM; however, this reduction was more distinct in DR (16%), followed by MR (1%), as shown in Fig. 4a. Similarly, the PR showed an increased level of 6% in POD activity compared to the control. We observed that the KNO_3_ treatment at 1.5 mM enhanced the activity of all attributes of Kentucky bluegrass leaves under drought stress (Fig. 4b). All three traits recorded lower activity under control conditions; however, the KNO_3_ treatment under drought stress led to the increase in POD activity up to 39%, 1% and 27% in PR, MR, and DR, respectively. The fertilization of NH_4_Cl and KNO_3_ at 15 mM remained less beneficial under drought stress than the control treatment (Fig. 4c,d). Both PR and MR showed less activity in response to the fertilization treatments under drought stress conditions, while DR was enhanced by 7% and 25% in NH_4_Cl and KNO_3_, respectively. An application of NH_4_Cl and KNO_3_ treatments at 30 mM enhanced the activity in MR by 28% and 21% under exposure to progressive drought stress, respectively, whereas PR and DR decreased in NH_4_Cl (19% and 1%, respectively) and only PR (0.5%) decreased in KNO_3_ compared to the control (Fig. 4e,f).

### Proline in the clonal ramets of Kentucky bluegrass under different nitrogen tracers and water supplies

**Figure 5:**
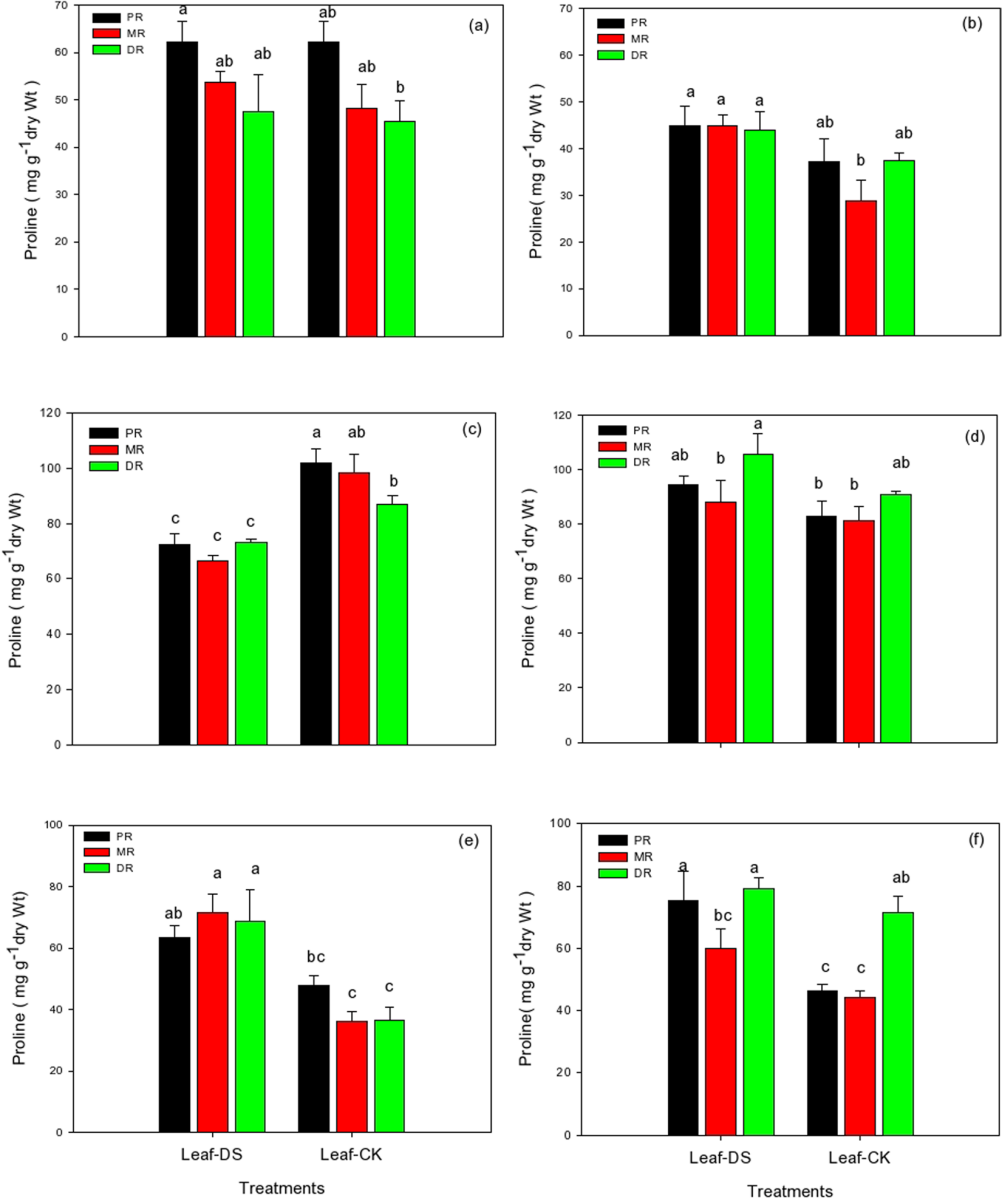
Proline contents in the clonal ramets of Kentucky bluegrass as affected by drought stress under three nitrogen isotopes at low, moderate and high rates. (**a**) ^15^NH_4_Cl at 1.5 mM, (**b)** K^15^NO_3_ at 1.5 mM, (**c**) ^15^NH_4_Cl at 15 mM, (**d**) K^15^NO_3_ at 15 mM, (**e**) ^15^NH_4_Cl at 30 mM, and (**f**) K^15^NO_3_ at 30 mM.

The proline content in PR, MR, and DR leaves exhibited at rapid rise under the different levels of nitrogen tracers; however, a decline was observed only at 15 mM of NH_4_Cl under drought stress (Fig. 5). NH_4_Cl treated plants at 1.5 mM increased the proline levels of PR, MR, and DR up to 18%, 10% and 4%, respectively. Among all three traits, PR performed well in response to fertilizer treatments under water stress conditions (Fig. 5a). Similar trends were observed using KNO_3_ at 1.5 mM, which led to a concomitant increase in proline content in all three attributes of Kentucky bluegrass leaves. Fertilization with KNO_3_ increased the proline content of MR leaves up 37%, followed by PR (17%) and then DR (15%), as shown in Fig. 5b. The NH_4_Cl treatment at 15 mM retarded proline content in all three traits in comparison with the control. Figure (5c) revealed that the NH_4_Cl treatment caused a decrease in MR proline content up to 33%, but this reduction was much lower in DR under drought stress conditions. Through the use of a moderate level of KNO_3_ at 15 mM, the proline contents of PR, MR, and DR were greater than the control plants. DR leaves demonstrated an increased level of proline content (14%) in response to KNO_3_ during water stress conditions (Fig. 5d). The proline content of all three traits was significantly enhanced with higher levels of the nitrogenous treatments of NH_4_Cl and KNO_3_ at 30 mM under drought stress conditions. More proline was synthesized in MR leaves (49%) in NH_4_Cl, followed by PR (39%) in the KNO_3_ treatments. On the other hand, PR (24%) in NH_4_Cl and DR (10%) in KNO_3_ were the least influenced by fertilization treatments with the induction of drought conditions (Fig. 5e,f).

### Changes in root and leaf nitrogen isotope abundance in the clonal ramets of Kentucky bluegrass under different nitrogen tracers and water supplies

**Figure 6:**
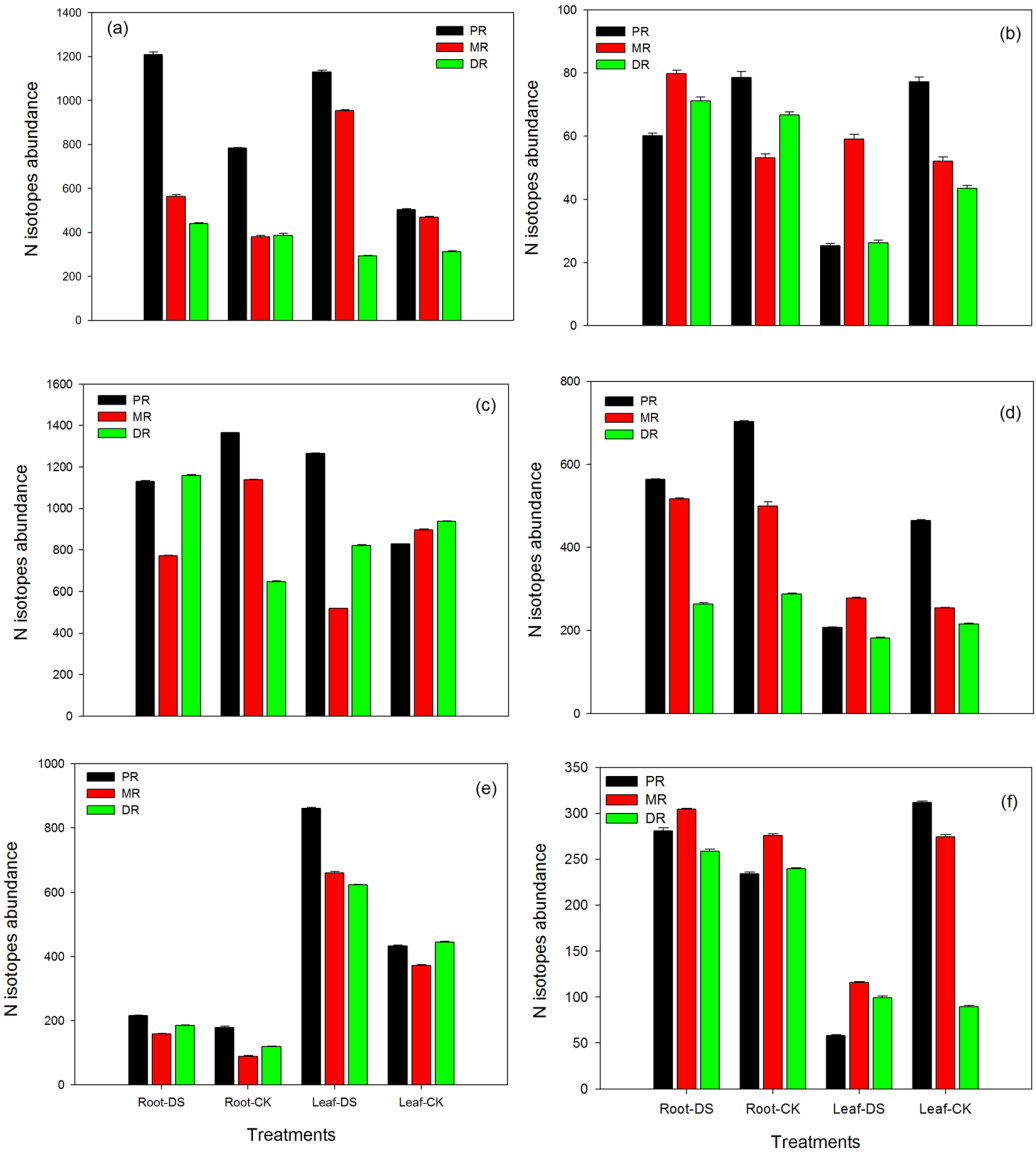
Nitrogen isotopes abundance in the roots and leaves of the clonal ramets of Kentucky bluegrass as affected by drought stress under three nitrogen isotopes at low, moderate and high rates. (**a**) ^15^NH_4_Cl at 1.5 mM, (**b**) K^15^NO_3_ at 1.5 mM, (**c**) ^15^NH_4_Cl at 15 mM, (**d**) K^15^NO_3_ at 15 mM, (**e**) ^15^NH_4_Cl at 30 mM, and (**f**) K^15^NO_3_ at 30 mM.

The nitrogen isotopes in both the roots and leaves of Kentucky bluegrass were significantly influenced by both fertilization treatments upon exposure to drought stress (Fig. 6). An application of NH_4_Cl and KNO_3_ at 1.5 mM was effective in enhancing the nitrogen isotopes in all three attributes. In the roots and leaves, PR demonstrated an increased level of nitrogen isotopes (35% and 55%, respectively) in response to NH_4_Cl, while with the KNO_3_ treatment, MR recorded a greater amount (33% and 12%, respectively) under water stress (Fig. 6a,b). An application of KNO_3_ at 1.5 mM decreased the nitrogen isotopes in PR roots up to 23%, while in the leaves of PR and DR, nitrogen isotopes were reduced up to the level of 67% and 40%, respectively. Compared to the control, an application at a moderate level of NH_4_Cl at 15 mM decreased nitrogen isotopes in PR and MR roots (17% and 32%, respectively), while in the leaves of MR and DR, nitrogen isotopes were reduced up to 42% and 12%, respectively, as shown in Fig. 6c. On the other hand, KNO_3_ at 15 mM was more effective in increasing (6% and 9%) the isotopes in the roots and leaves of MR. However, the isotope abundance in both the roots and leaves of PR and DR decreased (20% and 55%) and (8% and 16%) in response to KNO_3_ at 15 mM under drought stress, respectively (Fig. 6d). Nitrogen isotope abundance in the roots and leaves in all three traits were significantly influenced by the higher fertilization treatment of both NH_4_Cl and KNO_3_ at 30 mM under water stress conditions (Fig. 6e,f). Among the three traits, DR roots showed increased (36%) isotope abundance with NH_4_Cl at 30 mM, followed by MR (44%) and then PR (17%), while PR and MR leaves recorded an enhancement of 50% and 40% compared to the control, respectively. Applying KNO_3_ at 30 mM to plants during drought resulted in the increase of isotope abundance in the roots of all three attributes and only in DR leaves. PR and MR leaves had a reduction (81% and 58%, respectively) in isotope abundance during drought stress when KNO_3_ was applied at the rate of 30 mM to Kentucky bluegrass plants.

### Changes in root and leaf nitrogen content (%) in the clonal ramets of Kentucky bluegrass under different nitrogen tracers and water supplies

**Figure 7:**
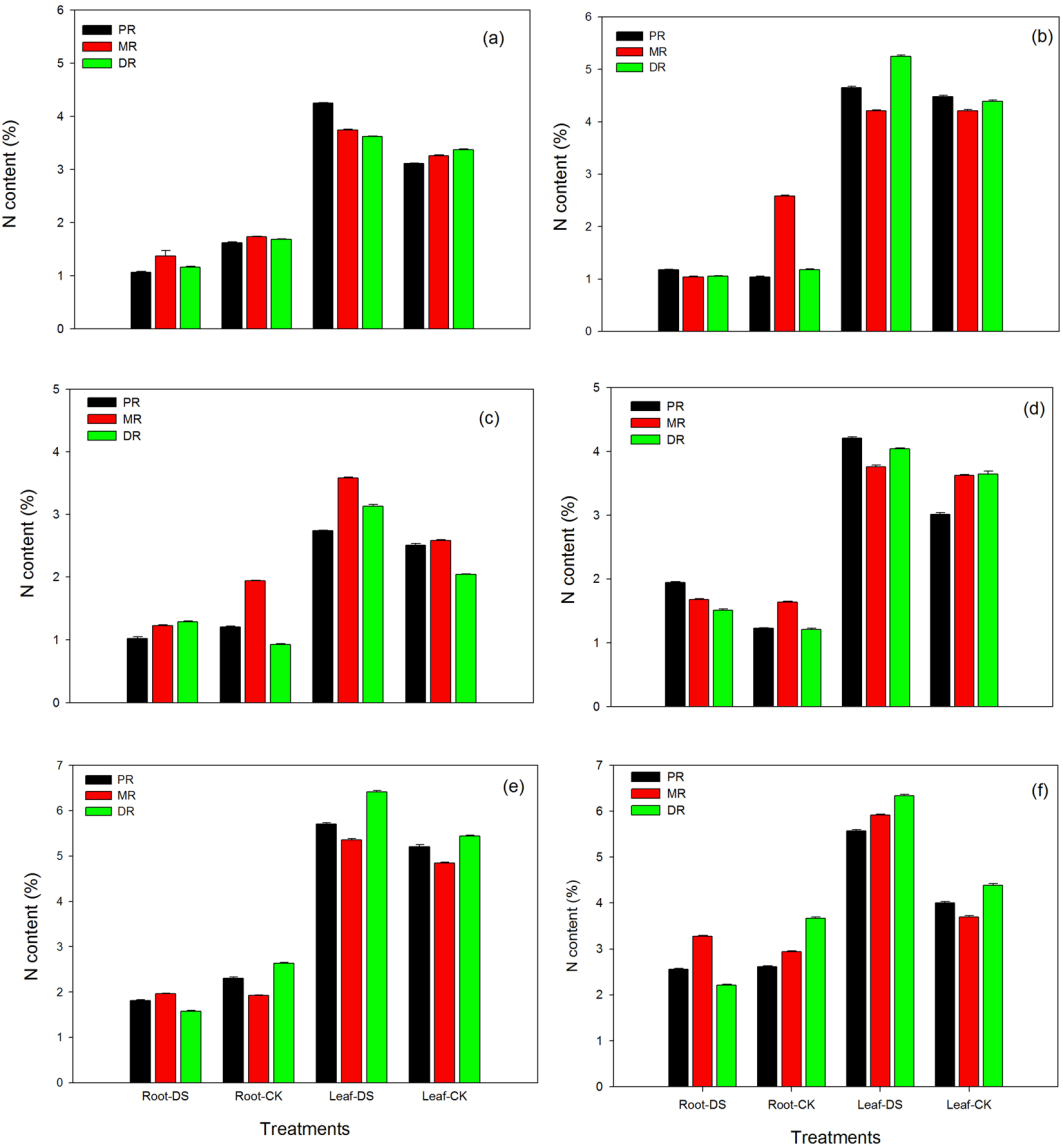
Nitrogen content in the roots and leaves of the clonal ramets of Kentucky bluegrass as affected by drought stress under three nitrogen isotopes at low, moderate and high rates. (**a**) ^15^NH_4_Cl at 1.5 mM, (**b**) K^15^NO_3_ at 1.5 mM, (**c**) ^15^NH_4_Cl at 15 mM, (**d**) K^15^NO_3_ at 15 mM, (**e**) ^15^NH_4_Cl at 30 mM, and (**f**) K^15^NO_3_ at 30 mM.

Upon exposure to nitrogenous fertilization under drought stress, the nitrogen content of PR, MR, and DR roots and leaves assuaged the adverse effects of water stress (Fig. 7). Maximum nitrogen content was observed in the roots of all three attributes under well-watered conditions compared to NH_4_Cl and KNO_3_ fertilization at 1.5 mM, and the maximum nitrogen content was observed in MR roots with the NH_4_Cl application between the NH_4_Cl and KNO_3_ treatments at 1.5 mM under drought stress conditions. A maximum reduction (35%) was recorded in PR roots, followed by DR (31%), in response to the NH_4_Cl treatment (Fig. 7a). However, NH_4_Cl fertilization at 1.5 mM significantly improved the nitrogen content of leaves. A maximum increase was recorded in PR (27%) and then in MR (13%), while the DR trait was increased only 6% under stress conditions. An application of KNO_3_ at 1.5 mM decreased nitrogen content in the roots of all three traits; however, this reduction was most pronounced in MR (60%), then PR (54%) and lastly DR (11%) relative to control conditions (Fig. 7b). On the other side, the KNO_3_ treatment increased the nitrogen content in the leaves of Kentucky bluegrass. A maximum increase (16%) was recorded in DR leaves, while the nitrogen content in MR leaves was not significantly affected by KNO_3_ fertilization. Maximum nitrogen content (28%) in DR roots was recorded with an application of NH_4_Cl at 15 mM; nevertheless, the level of nitrogen content decreased in PR (16%) and MR (37%) under stress conditions. The fertilization of NH_4_Cl remained beneficial, and NH_4_Cl at 15 mM recorded 8%, 28% and 35% in the nitrogen content of PR, MR and DR, respectively (Fig. 7c). Nitrogen content significantly increased in both the roots and leaves of bluegrass plants grown in stressful conditions with a KNO_3_ application at 15 mM. A maximum nitrogen content was recorded in both the roots and leaves (37% and 29%, respectively) of PR, while the MR trait showed the lowest (2% and 4%, respectively) content (Fig. 7d).The application of a high level of NH_4_Cl at 30 mM did not increase the nitrogen content in the roots of PR and DR, while only a 2% enhancement was recorded in MR roots. There was a maximum reduction of 40% in DR roots, while only a 22% reduction was observed in PR in response to the NH_4_Cl treatment. An application of NH_4_Cl fertilization remained beneficial in the leaves, as this treatment enhanced the nitrogen content of all three traits. The DR leaves demonstrated a maximum nitrogen content (15%), followed by MR (10%) and PR (9%), compared to the control (Fig. 7e). The root contents of PR and DR showed a decrease in nitrogen content in response to KNO_3_ at 30 mM; however, MR roots demonstrated a positive response to the fertilization treatment. In response to KNO_3_ at 30 mM, a maximum reduction of 40% in DR roots and a 10% enhancement in MR were recorded under drought conditions. On the other hand, the leaves of all traits recorded a maximum amount of nitrogen content in the stressed environment. MR leaves (38%) showed the highest nitrogen content, followed by DR (31%) and PR (28%), relative to the control (Fig. 7f).

### Changes in relative water content (%) in the roots and leaf of the clonal ramets of Kentucky bluegrass under different nitrogen tracers and water supplies

**Figure 8:**
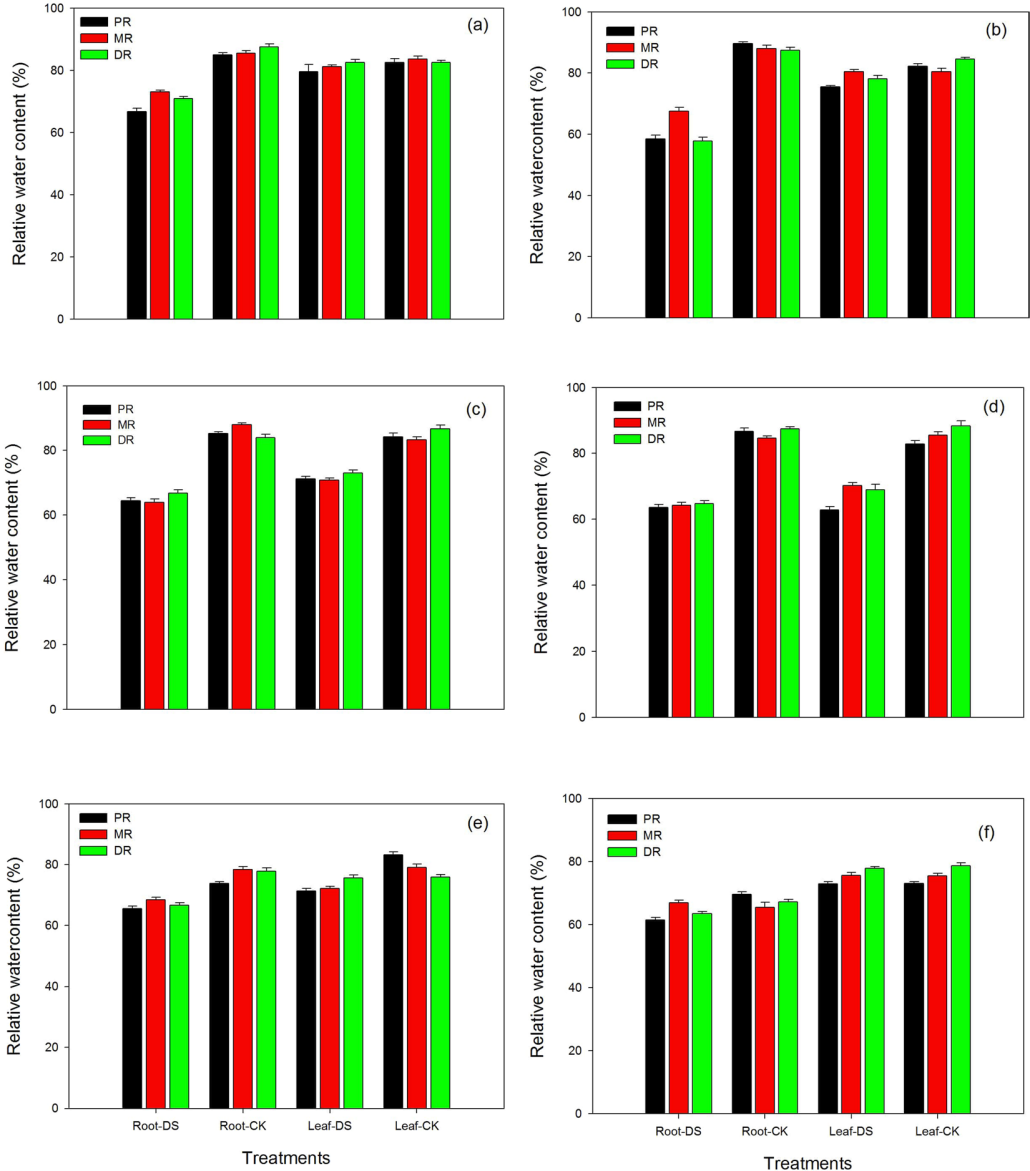
Relative water content in the roots and leaves of the clonal ramets of Kentucky bluegrass as affected by drought stress under three nitrogen isotopes at low, moderate and high rates. (**a**) ^15^NH_4_Cl at 1.5 mM, (**b**) K^15^NO_3_ at 1.5 mM, (**c**) ^15^NH_4_Cl at 15 mM, (**d**) K^15^NO_3_ at 15 mM, (**e**) ^15^NH_4_Cl at 30 mM, and (**f**) K^15^NO_3_ at 30 mM.

We observed that the leaf water content of all three traits in response to nitrogenous fertilizer treatments were significantly influenced by drought stress. Maximum leaf water content was observed in both the roots and leaves of all three traits grown in well-watered conditions (Fig. 8). Under stress conditions, a 22%, 14% and 19% reduction was observed in PR, MR and DR roots, respectively, in response to NH_4_Cl at 1.5 mM. Among the control plants, DR roots showed higher amounts of water content than MR and PR. Similarly, the leaves of stress grown plants had lower water contents than the control. There was a maximum reduction of 4% in PR leaves, followed by MR (3%), in response to the NH_4_Cl treatment. However, water content increased only 0.12% in DR leaves, as shown in Fig. 8a. The water content of both the roots and leaves of PR, MR and DR decreased in stress conditions under KNO_3_ at 1.5 mM relative to the control treatment. With the application of KNO_3_ in drought, a maximum reduction of 35%, 23% and 34% was recorded in PR, MR and DR, respectively. However, in leaf, a minimum reduction of water content was noted compared to the roots. A maximum reduction of 8% was observed in both PR and DR, while in MR, the water content was similar to the control (Fig. 8b). The data in Fig. 8c indicated that the water contents of MR roots (27%) decreased while DR (20%) was the least affected by the application of NH_4_Cl at 15 mM. However, water contents in the leaves declined in stress conditions but not abruptly in the roots. A reduction in the range of 15–16% was noted in the leaves of all three traits of Kentucky bluegrass with NH_4_Cl applications. The fertilization of KNO_3_ at a moderate level of 15 mM recorded a reduction range of 24 to 27% and 18 to 24% in the roots and leaves, respectively, in the PR, MR, and DR traits. Under stress conditions, a maximum reduction was recorded in both the roots and leaves of PR plants, as shown in Fig. 8d. At 30 mM, the NH_4_Cl application exhibited an 11 to 14% and 0.5 to 14% decline in water content compared to the control. Furthermore, there was a maximum reduction in DR roots and PR leaves in response to NH_4_Cl under drought conditions (Fig. 8e). The water contents of both roots and roots were also considerably decreased in Kentucky bluegrass with an application of KNO_3_ at 30 mM and upon the exposure of drought stress. A reduction of 0.4 to 11% and 0.1 to 1% in the roots and leaves, respectively, of PR, MR and DR traits were noted under stress conditions (Fig. 8f).

### Changes in water potential (-Mpa) in the roots and leaf of the clonal ramets of Kentucky bluegrass under different nitrogen tracers and water supplies

**Figure 9:**
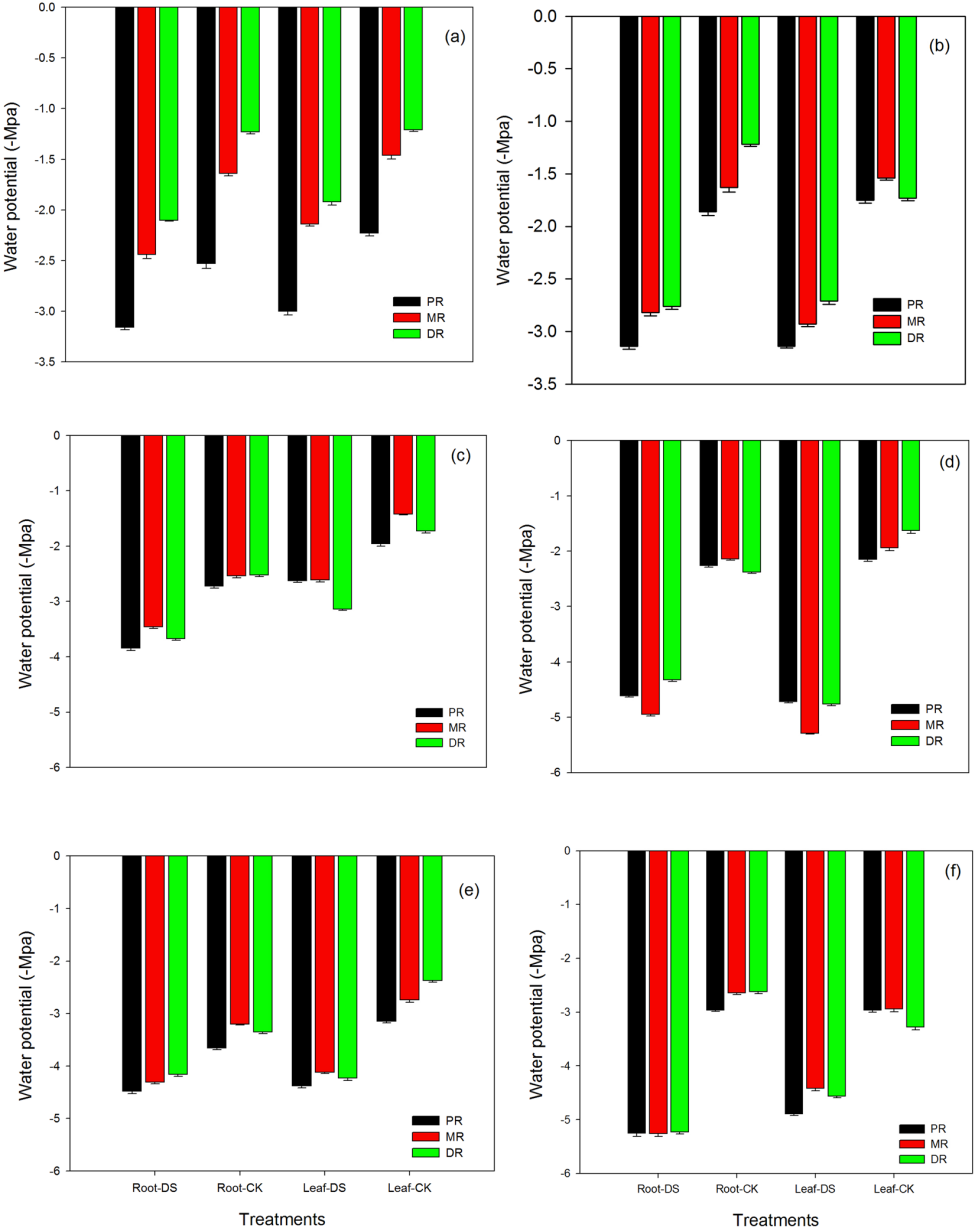
Water potential in the roots and leaves of the clonal ramets of Kentucky bluegrass as affected by drought stress under three nitrogen isotopes at low, moderate and high rates. **(a**) ^15^NH_4_Cl at 1.5 mM, (**b)** K^15^NO_3_ at 1.5 mM, (**c**) ^15^NH_4_Cl at 15 mM, (**d**) K^15^NO_3_ at 15 mM, (**e**) ^15^NH_4_Cl at 30 mM, and (**f**) K^15^NO_3_ at 30 mM.

The fertilization treatment under drought stress conditions consistently and significantly (P ≤ 0.05) reduced root and leaf water potential in PR, MR and DR traits in comparison to the controls of Kentucky bluegrass (Fig. 9). Among the traits we examined, drought stress decreased the root water potential of all three traits in the range of 20 to 41% under NH_4_Cl at 1.5 mM. The water potential of DR roots was severely influenced by stress conditions, followed by MR, compared to the control treatments. Nonetheless, the trend of declination in water potential was also recorded in the leaves of Kentucky bluegrass. A significant reduction of 36%, 32% and 37% in PR, MR and DR leaves, respectively, was noted at 1.5 mM of the NH_4_Cl application, as shown in Fig. 9a. The DR leaves showed the maximum amount of water potential among the control treatments. Next, we addressed the influence of progressive drought and KNO_3_ at 1.5 mM on the roots and leaves of all three attributes. The water potential of both the roots and leaves in all traits were mostly suppressed by prolonged water stress at 1.5 mM of the KNO_3_ application. Therefore, 41 to 56% and 36 to 47% reductions in the water potential of the roots and leaves, respectively, were recorded in PR, MR and DR of Kentucky bluegrass. DR roots and MR leaves exhibited maximum water potential in the control plants compared to the rest of the treatments (Fig. 9b). Applying NH_4_Cl at 15 mM to drought plants resulted in decreased water potential of both the roots and leaves of all three traits of Kentucky bluegrass. At 15 mM of NH_4_Cl fertilization in drought conditions, a considerable decline in the range of 27–31% and 25–46% was recorded in the roots and leaves, respectively, of PR, MR and DR compared to the control plants. However, the data pertaining to water potential (Fig. 9c) indicated that DR roots and MR leaves of the control plants showed maximum potential. The same trend was also observed with a moderate application of KNO_3_ treatment at 15 mM under water stress conditions; however, the reduction was more severe than with NH_4_Cl fertilization. It was noted that there was a 45 to 57% and 54 to 66% reduction in the water potential of the roots and leaves, respectively, of all three attributes under a KNO_3_ application at a moderate rate (Fig. 9d). Likewise, the water potential of roots and leaves declined by 18 to 26% and 28 to 44%, respectively, in response to a high level of NH_4_Cl at 30 mM under drought conditions. However, MR roots and DR leaves recorded maximum water potential among all attributes of the control treatment (Fig. 9e). In KNO_3_ fertilization at 30 mM, the water potential of all the three traits was drastically decreased in comparison with the NH_4_Cl application. A substantial reduction of 44 to 50% and 28 to 39% under KNO_3_ fertilization was recorded in the roots and leaves of PR, MR and DR traits grown in stressful conditions. Among all attributes under drought conditions, the water potential of PR roots and leaves was significantly affected, as shown in Fig. 9f.

## Discussion

The physiological integration is one of the physiology characteristics and one of the important mechanisms in the growth, development and adaptation of clonal plants. Resources translocate or redistribute among ramets increases clonal growth and enhance the ability to adapt adverse environments. The present study assessed three ramet types of Kentucky bluegrass for their physiological and biochemical responses to nitrogen isotopes labelled ^15^NH_4_Cl and K^15^NO_3_ under prolonged drought conditions. Under a drought stress environment, nutrient absorption and water uptake are reduced, which led to decreased morphological, physiological and biochemical attributes of the plants. Consequently, plant performance was influenced^39^. Under N nutrition, there was an improvement in the SOD, POD, and catalase activity systems in the water-stressed plants, which could be the strategy for enhancing the drought resistance ability. In this study, N could have contributed to the defence of photosynthetic organs, and these findings are consistent with Saneoka *et al*.^40^ who recommended that N nutrition played a pivotal role in the drought tolerance of bentgrass by stopping cell membrane damage and enhancing osmoregulation (Figs. 2–4). In plants, a variety of antioxidant enzymes, lipids and water soluble molecules play vital roles in scavenging reactive oxygen species. Of these, antioxidant enzymes are considered the most effectual in reducing oxidative damage^7,41,42^. The results depicted that an application of nitrogenous fertilizer improved the drought tolerance in all three ramet types due to enhanced the functions of cellular antioxidants detoxification and osmoprotection, which were taken as the main indicators of stress tolerance (Figs. 2–4). NH_4_Cl and KNO_3_ tracers could enhance the performance of protective enzymes as observed in our study, and these antioxidant enzymes could alleviate the harmful influence of ROS (reactive oxygen species) on plants and retained a balance between their production and their removal during stressful conditions^7^ In the present study, increased levels of antioxidant activities with nitrogen isotope applications helped the three ramets of Kentucky bluegrass overcome injuries to the tissues and decreased cellular toxic levels produced under prolonged water stress environments. Basu *et al*.^43^ also observed that indica rice (*Oryza sativa indica*) tolerance to drought stress was associated with the enhanced capability of antioxidant enzymes to decrease the negative effects induced by drought stress. However, there was generally difference observed between the integration of SOD, POD and CAT in PR, MR and DR of Kentucky bluegrass, which confirmed the physiological integration did exist in SOD, POD and CAT activities between ramets, and in most cases, the MR performed more active than PR and DR indicated that MR take more responsibility to balance the vitality of other ramets under drought conditions.

Proline is considered one of the most significant osmolytes, which assists in promoting water retention and reducing the damaging effects of water stress in plants^44^. During adverse environmental conditions, it deposits quickly and more frequently as compare to any other amino acid^45^. It could play a vital role in stress-resistance mechanisms by performing as an osmoprotectant, assisting osmoregulation, the stabilization of protein-synthesis machinery, and the regulation of cytosolic acidity^46^. We observed increased levels of proline in all three ramet traits under drought stress in response to nitrogenous fertilizer (Fig. 5); however, this enhancement was less pronounced in control plants and could be due to increased cytosolic concentrations of osmolytes in drought conditions, which not only assist in maintaining the tissues but also takes part in osmoregulation^7,47^.

The accumulation of N isotope abundances increased with the application of NH_4_Cl in both the roots and leaves however, KNO_3_ only enhanced it in root portion of all three ramets, which facilitated plants to perform better during drought stress (Fig. 6). Yoneyama *et al*.^33^ reported that the δ^15^N values in the xylem and plant tissues are associated with acquired N, and the value can be altered by N metabolism. Our results also indicated that drought stress conditions inhibited nitrogen fixation and could be due to the fact that nitrogenase is more sensitive than nitrate reductase. In the present study, application of NH_4_Cl and KNO_3_ under drought stress enhanced the nitrogen contents more pronouncedly in the leaves of all three ramets compared to control conditions; however, in root portion the augmentation was not so high under drought stress (Fig. 7). The differences in nitrogen content between three ramets along with the nitrogen distribution indicated that the nitrogen were integrated. Nitrogen is a significant constituent of Chl, proteins and Rubisco, which influences plant metabolism during water stress conditions. Grassi and Magnani^48^ suggested that adequate N increased the resurgence of photosynthetic biochemistry, and its slow recovery was noted in those conditions where plants were exposed to severe drought stress conditions under limited N supplies. Therefore, better nitrogen nutrition could alleviate the damaging effects of drought stress by sustaining the metabolic activities even at low tissue water potential, as recorded in our study.

Plant-water relations in response to N nutrition in Kentucky bluegrass roots and leaves were also disturbed under water stress conditions (Figs. 8, 9); however, moderate and high levels of NH_4_Cl and KNO_3_ improved these relations, so our results recommended that plant-water relations (both relative water content and water potential) may serve as an indicator of plant water status, and three ramets capability to sustain sufficient water status develops water stress adaptability by increasing drought resistance. Relatively, the water content of DR was higher than PR and DR, which indicated that the water was integrated by three ramets and tended to transport to young plants under different nitrogen drought conditions. Under different abiotic stresses, reduced water potential are the major causes of loss in dry matter production and the other attributes of plants^49^. By maintaining water potential, the accretion of compatible osmolytes engaged in osmoregulation allowed supplementary water to be taken up from the environment, thus buffering the instant influence of water scarcity within the organism^7,50–52^.

## Conclusion

To the best of our knowledge, using the nitrogen isotopes to quantitatively infer the physiological activities responding to nitrogen and drought in Kentucky bluegrass within the clonal ramets has not been addressed before. The present results clearly show that it is effectively using nitrogen isotopes to infer the impact of drought stress and nitrogen on the physiological attributes of Kentucky bluegrass clonal remats. The antioxidant activities and proline contents of the ramets in Kentucky bluegrass were observed to be correlated with N-mediated and physiological integration. The SOD, POD, CAT activities and free proline content in the ramet of MR performed more active than PR and DR indicated that MR take more responsibility to balance the vitality of other ramets under drought conditions. Nitrogen concentration with 30 mM caused significant down-regulation of *PpGS2* expression, but it was slightly up-regulated after imposed drought. Plant-water relations in response to nitrogen in Kentucky bluegrass were also integrated by three ramets and tended to transport to young plants under different drought and nitrogen conditions. In addition, nitrogen isotope abundance was enhanced in both the roots and leaves of the three ramets of Kentucky bluegrass with the application of ^15^NH_4_Cl, however, K^15^NO_3_ only enhanced in root portion. The nitrogen content was more pronounced in leaves compared to roots under both drought and control conditions. Nitrogen status of Kentucky bluegrass implied that nitrogen assimilation and allocation was integrated with a certain orientation and location characteristics within the remats.
